# Real-Time Tracking Target System Based on Kernelized Correlation Filter in Complicated Areas

**DOI:** 10.3390/s24206600

**Published:** 2024-10-13

**Authors:** Abdel Hamid Mbouombouo Mboungam, Yongfeng Zhi, Cedric Karel Fonzeu Monguen

**Affiliations:** 1 School of Automation, Northwestern Polytechnical University, Xi’an 710002, China; yongfeng@nwpu.edu.cn; 2 Institute for Chemical Technology and Polymer Chemistry, Karlsruhe Institute of Technology (KIT), Engesserstr. 20, 76131 Karlsruhe, Germany; cedric.monguen@kit.edu

**Keywords:** object detection, real-time object tracking, kernelized correlation filter, model drift, variance classifier

## Abstract

The achievement of rapid and reliable image object tracking has long been crucial and challenging for the advancement of image-guided technology. This study investigates real-time object tracking by offering an image target based on nuclear correlation tracking and detection methods to address the challenge of real-time target tracking in complicated environments. In the tracking process, the nuclear-related tracking algorithm can effectively balance the tracking performance and running speed. However, the target tracking process also faces challenges such as model drift, the inability to handle target scale transformation, and target length. In order to propose a solution, this work is organized around the following main points: this study dedicates its first part to the research on kernelized correlation filters (KCFs), encompassing model training, object identification, and a dense sampling strategy based on a circulant matrix. This work developed a scale pyramid searching approach to address the shortcoming that a KCF cannot forecast the target scale. The tracker was expanded in two stages: the first stage output the target’s two-dimensional coordinate location, and the second stage created the scale pyramid to identify the optimal target scale. Experiments show that this approach is capable of resolving the target size variation problem. The second part improved the KCF in two ways to meet the demands of a long-term object tracking task. This article introduces the initial object model, which effectively suppresses model drift. Secondly, an object detection module is implemented, and if the tracking module fails, the algorithm is redirected to the object detection module. The target detection module utilizes two detectors, a variance classifier and a KCF. Finally, this work includes trials on object tracking experiments and subsequent analysis of the results. Initially, this research provides a tracking algorithm assessment system, including an assessment methodology and the collection of test videos, which helped us to determine that the suggested technique outperforms the KCF tracking method. Additionally, the implementation of an evaluation system allows for an objective comparison of the proposed algorithm with other prominent tracking methods. We found that the suggested method outperforms others in terms of its accuracy and resilience.

## 1. Introduction

In recent years, there has been increasing interest in real-time tracking and target identification across several contemporary applications. This can be attributed to the widespread use of surveillance cameras and their increasing deployment, especially in security and surveillance domains [1].

Although object tracking is commonly used in computer vision research to refer to monitoring a single item in a video, Multi-Target Tracking (MTT) is something that people do in the real world.

In computer vision for MTT, two techniques are employed. The first creates several instances of a single-object tracker (SOT) and assigns each one to a different target. The same approach is utilized to track numerous items in this case, and cognitive neuroscience studies of single-object tracking can be useful. The second technique creates an MTT algorithm that can monitor many objects at the same time. This technique is helpful because it may benefit from the system’s shared information, which is important for tracking individuals and dealing with issues. In general, MTT algorithms in computer vision, especially brain-inspired algorithms, are still far from the precise processes used by the brain. However, there is much more to be learned from MTT and cognitive neuroscience.

Regarding the effect of semantic information, MTT is facilitated by the semantic distinction between target and distractor categories. This facilitation is due to the categorical separation of objectives and distractors, which is supported by four processes. For starters, any visual contrast between objectives and distractions aids in tracking. Second, semantic distinctions between targets and distractors necessitate the attentional system selecting a different method for allocating attention to objects, therefore facilitating tracking. Third, category information may be kept in visual working memory, improving mistake recovery. Fourth, there is a system that categorizes information, making it simpler to monitor targets amid distractors belonging to different categories. 

For the effect of surface features, color and form are the most immediately perceivable surface characteristics that aid an observer in distinguishing between items. One research work looked into whether surface-feature information helps MTT or whether monitoring numerous objects is dependent only on spatiotemporal information [2]. Surface characteristics such as color and form are stored in the brain’s early visual processing regions [3]. These characteristics, according to prior studies, definitely impact the processing of targets and distractors during MTT and aid tracking. MTT methods, on the other hand, lack generality and are biased toward finding the optimal feature set for a single application.

In terms of the effect of motion features and depth in MTT, motion information plays a crucial role [4]. A task that introduces motion into the texture of each item and the backdrop is meant to examine the influence of motion information on visual attention via MTT [5]. 

In MTT tasks, in addition to intrinsic motion, an item’s speed and direction of movement are often given at random to push the object to have different motion trajectories. In other research, velocity is assumed to be constant in order to disregard the influence of acceleration [6].

Among the various methods used for target tracking, the correlation filtering approach stands out due to its efficiency and robustness [7]. A real-time target tracking system based on kernel correlation filtering is a computer vision system that uses kernel correlation filters to track targets in real time. Unlike deep learning, which relies on large training datasets, correlation filter-based trackers such as kernelized correlation filters (KCFs) utilize the implicit properties of tracked images, such as their circular configuration, for real-time training [8]. This system begins by detecting the target in the initial frame of the video sequence and subsequently tracks the target in subsequent frames using a kernel correlation filter [9]. 

The computational efficiency of KCFs, which makes them well suited to low-power heterogeneous computational processing technologies, lies in their ability to compute data in a high-dimensional feature space without explicitly performing computations in this space [10]. A kernel correlation filter is a mathematical algorithm that uses a kernel function to map the input image to a high-dimensional space. The filter calculates the correlation between the target template and the input image in this high-dimensional space, enabling it to track the target even as scale, rotation, and lighting conditions change. A real-time tracking target system based on kernel correlation filtering offers several advantages over other tracking systems. It is computationally efficient and enables real-time tracking of targets. Furthermore, it is safe when changes in a target’s appearance occur, making it suitable for tracking targets in complex situations [11]. Kernelized correlation filters (KCFs) have gained significant popularity due to their satisfactory speed and accuracy since their inception. They are particularly well suited to target tracking systems with high real-time requirements. However, they lack the ability to detect tracking failures, so they are not suitable for long-term target tracking. Based on previous research, we propose an improved KCF that meets the requirements of long-term target tracking. Firstly, we introduce a confidence mechanism to evaluate the target tracking results and determine the tracking status. Secondly, we design a tracking model update strategy to reduce the interference from background information, thereby enhancing the robustness of the algorithm.

Several studies on real-time detection and tracking algorithms have been conducted in the past. Abdulghafoor and Abdullah (2022) developed a novel real-time framework for detecting and tracking multiple objects, addressing various challenges [1]. Afterwards, Guoqing Shi et al. (2022) conducted research to enhance the kernel correlation filtering algorithm by incorporating the Kalman filter [12]. Subsequently, Sun et al. (2023) introduced a target tracking approach utilizing a kernelized correlation filter in conjunction with MWIR and SWIR sensors [13]. Following this, Feng and Wang (2023) proposed a model-adaptive updating kernel correlation filter tracker that incorporated deep CNN features [14]. Each of these studies presented distinct methodologies in order to identify the most effective techniques that yielded optimal results.

The major focus of this research is to investigate the target tracking approach in the context of high real-time requirements while addressing the challenge of tracking robustness. The second part provides an overview of the necessary theoretical foundation. We will delve into the kernel-based tracking approach and explore methods to improve the scale estimation. Finally, this research presents the experimental findings and a thorough analysis of the tracking algorithms. This includes the introduction of a tracking algorithm evaluation index system, a comparative analysis of the improved algorithm and the core experimental results of related tracking algorithms, and a comparison between the algorithm developed in this study and other prominent tracking algorithms.

## 2. Methodology and Development of the Proposed Tracking Method

### 2.1. Typical Links of Target Tracking

**Figure 1:**
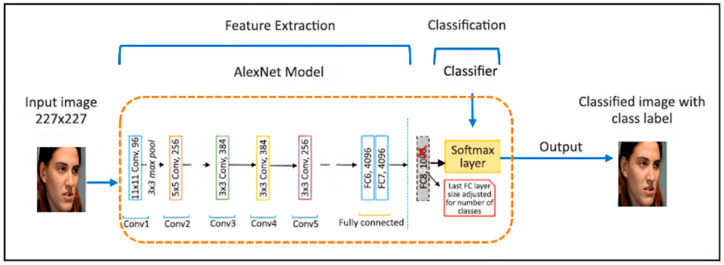
Typical target tracker composition [16].

Image target tracking means that after manually specifying a target, the image processing system gives the target status information for each frame of the image in the field of view at the time. As shown in a typical target tracker [15], it can be divided into the following four parts: a search mechanism, feature extraction, a target classifier, and a model update mechanism. Sometimes, in order to enhance the model’s robustness, some researchers have suggested tracking algorithms that integrate multiple features or multiple trackers. The following Figure 1 describe a typical target tracker composition:

### 2.2. Basic Theory 

#### 2.2.1. The Nuclear Method

Most real-world models are nonlinear, and the linear mode is a specific aspect of nonlinear models. In exceptional cases, if the target classifier in the target tracking field employs a nonlinear model, its classification impact will be superior to that of a linear model. There are two approaches to dealing with the problem of nonlinear pattern recognition: one is to directly locate nonlinear patterns in the data linear model; another is to translate the original data into a high-dimensional feature space using a mapping function and then use linear algorithms to discover the linear patterns in this high-dimensional space. Human research on linear problems is well established, the computation efficiency in linear problems is high, and the application of nonlinear models requires a more accurate understanding of the data beforehand.

**Figure 2:**
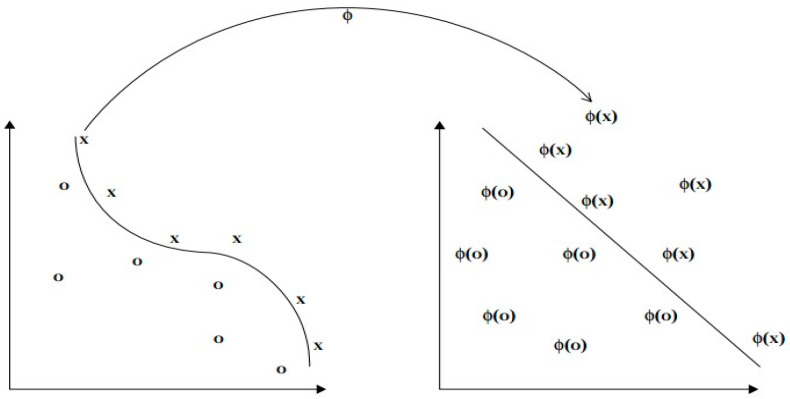
Function mapping the original linearly inseparable data to the linearly separable high-dimensional space [12].

Because these models’ normalizing capacity is limited, the second method of thinking is commonly utilized when dealing with nonlinear models. As demonstrated in the following figure (see Figure 2), after using the mapping function to move the original data from the original space to the high-dimensional space, the data in the high-dimensional space are linearly separable. The regression problem may now be identified using the algorithm to solve.

However, if this method is used directly, there are two issues to address. One approach is to work directly in the high-dimensional feature space; avoiding the curse of dimensionality is one option; the other is mapping each sample point to a high-dimensional space and then solving this space. This procedure is time-consuming, and the calculating efficiency is low.

**Figure 3:**
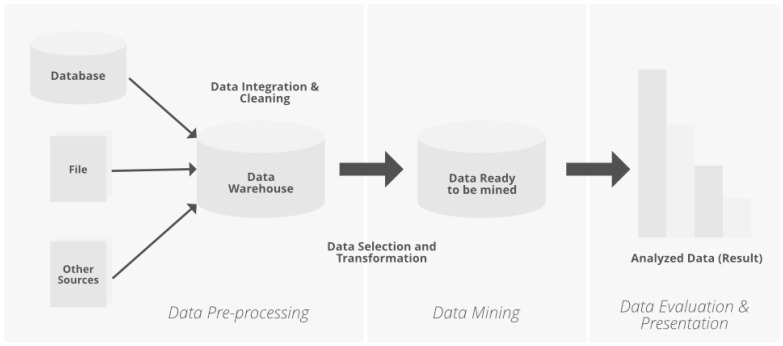
PA algorithm method’s flow.

We can convert the original ridge regression into its dual form, where the training process only needs to collect the inner product of the sample, and the prediction process only needs to compute the training sample. The inner product does not require direct knowledge of the sample and the regression variable values. As a result, we cannot display the mapping function, but we can easily address the ridge regression problem by demanding only its inner product. The following Figure 3 illustrates an analysis of the flow of the processing mode in the nuclear method, where the data processing procedure utilizes the kernel function to generate the kernel matrix. We then process the kernel matrix using the pattern analysis method (PA algorithm) to obtain a pattern function. Lastly, the model formula function is utilized to handle new cases effectively.

#### 2.2.2. The Circulant Matrix

The circulant matrix’s simplicity and other outstanding properties make it frequently employed in a variety of professions. As an example of working process applications, we can find the limits of linear time-invariant systems in signal processing [3], sparse signal reconstruction in compressed sensing [4], and deblurring methods in image processing [5]. Similarly, in machine vision, the circulant matrix may be utilized to describe the training dataset [17].

**Figure 4:**
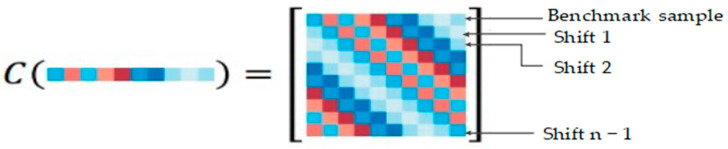
Cycle shift (one dimension).

This section starts with a one-dimensional vector to show the circulant matrix and then expands to two-dimensional. For vectors $x={\left[x_{1},x_{2},...,x_{n}\right]}_{1\;2}$, you can use it to generate a circulant matrix *C*(*x*). The following Figure 4 shows the cycle shift from a one dimensional vector.

(1) $$C\left(x\right)=\left[\begin{array}{ccc} x_{1} & \cdots & x_{n}\\ \vdots & \ddots & \vdots \\ x_{2} & \cdots & x_{1} \end{array}\right]$$

We can use a cyclic shift operator to generate a one-dimensional image offset transformation, and this operator can be used as follows:

The permutation matrix P can be represented by

(2) $$P=\left[\begin{array}{ccc} 0 & \cdots & 1\\ \vdots & \ddots & \vdots \\ 0 & \cdots & 0 \end{array}\right]$$

Product $P^{x}={\left[X_{n},X_{1},X_{2},\ldots ,X_{n-1}\right]}^{T}$, with x moving an element to create a small offset due to the cycle

(3) $$\left\{P^{u}\;x\left|u=0,\ldots \ldots ,n-1\right.\right\}$$

All the offset vectors in the composed set can be obtained by the following formula:

(4) $$C\left(x\right)=\begin{array}{c} P^{0}X^{T}\\ P^{1}X^{T}\\ P^{n}X^{T} \end{array}$$

Because of the cyclic properties, we can consider the first half of the elements in the set to be x in the square, whereas the latter half is thought to be formed by shifting in the negative direction. We then construct a circulant matrix using the loop offset approach. After locating the goal, shifting according to varied offsets can yield all the shifted samples relative to the reference sample. We determine the sample’s regression value and then use these samples to train the classifier.

On the outside, the edges are not smooth because of the cyclic shift, and the cosine function may be employed to reduce the boundary effect (as shown in the figure). As a result, we simply represent the sample set using the circulant matrix, as shown below. The most significant advantage of employing the circulant matrix to enhance the sample set is that it can be readily diagonalized, which dramatically accelerates the model training and prediction speed.

**Figure 5:**
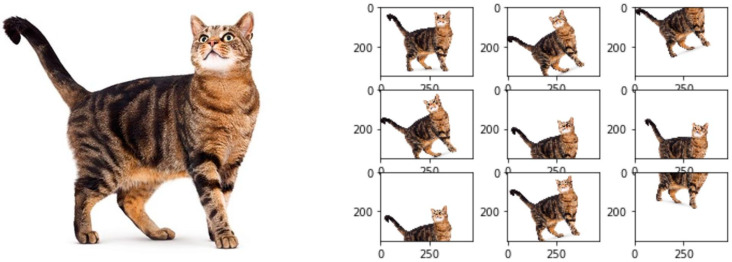
Cyclic shifting training samples.

The following Figure 5 shows cyclic shifting that generates training samples (the left picture is a two-dimensional cyclic sample generated by shifting, and the right picture is the corresponding sample).

### 2.3. Tracking Based on Correlation Filtering and Its Improvements

Initially, researchers used correlation filters in signal processing, but they later extended their use to pattern matching. Image target tracking involves a significant amount of computational work, as it necessitates the identification of the target phase among numerous candidate locations with the highest likelihood. Early target tracking algorithms often lacked real-time performance and tracking capabilities. The complexity of the procedure in particular restricts the target tracking method in an embedded program. 

Kernel-related filter tracking begins with ridge regression based on the kernel technique, which uses the circulant matrix as the training sample set and the test sample set. Due to the circulant matrix’s nature, the final model’s training, and the sample detection process, we efficiently perform the Fourier transform in the frequency domain, ensuring a balance between the computation efficiency and tracking performance. First and foremost, this is connected to filtering, and then nuclear-related filtering tracking is performed. The target scale cannot be approximated, as nuclear correlation filter tracking solely pertains to the testing location.

#### 2.3.1. Correlation Filtering

Given a test image *I* and a reference image *T*, $T\left(2N+1\right)\times T\left(2N+1\right)$ and its related operations are defined as defined symbols here for correlation operations.

(5) $$T\times I\left(x,y\right)=\sum_{i=-N}^{N}\sum_{i=-N}^{N}T\;\left(i,j\right)\times I\;\left(i+x,\;j+y\right),\;x,y=1,2,\;\ldots ,\;M$$

The next section examines how to use correlation filtering for template matching. The aim of template matching is to put an image to the test. *T* is a reference image, the most advantageous match point. To discover the optimum matching point, you must first develop a way to quantify *I*. *T* is a sub-image and a reference image; finally, *xy* should be iterated over.

(6) $$D(x,y)=\sum_{i=-N}^{N}\sum_{i=-N}^{N}{\left(T\;\left(i,j\right)-I\left(i+x,j+y\right)\right)}^{2}$$

According to Formula (6)’s definition of *I*(*x*, *y*), in $\left(2N+1\right)\times \left(2N+1\right),$ the Euclidean distance from the sub-image and reference image T is used as the degree of mismatch; template matching is to find *D*(*x*, *y*), with (*x*, *y*) being the smallest position.

(7) $$D(x,y)=\sum_{i=-N}^{N}\sum_{i=-N}^{N}T\;{\left(i,j\right)}^{2}+\sum_{i=-N}^{N}\sum_{i=-N}^{N}I\;{\left(i+x,\;j+y\right)}^{2}-2\sum_{i=-N}^{N}\sum_{i=-N}^{N}\left(T\;\left(i,j\right)\times I\;\left(i+x,\;j+y\right)\right)$$

Equation (7) simply connects the first component to the reference picture, ignoring the coordinate’s position. The second item is the sum of the squares of the gray values of pixels in the filter’s coverage region. The final component consists of two negative test pictures and the parameters associated with these pictures. If there is no significant change in the level of gray in the test image, we can discard the second term and solely connect (*i*, *j*) to the third term. The Euclidean distance decreases with a higher correlation value between the test and reference images. In many cases, we use the assumption that the level of gray in the test image does not fluctuate significantly.

(8) $$C\left(x,y\right)=\frac{\sum_{i=-N}^{N}\sum_{i=-N}^{N}\left(T\;\left(i,j\right)\times I\;\left(i+x,\;j+y\right)\right)}{\sqrt{\sum_{i=-N}^{N}\sum_{i=-N}^{N}(T\;\left(i,j\right)2+\sum_{i=-N}^{N}\sum_{i=-N}^{N}I{\left(i+x,\;j+y\right)}^{2}}}$$

According to the results of the preceding study, related processes can easily fulfill the template matching task, causing the associated templates to rotate 180°. In the future, the correlation and convolution operations will be the same; therefore, the convolution theorem can be utilized to adjust the correlation. Switching to the frequency domain significantly accelerates the calculations.

#### 2.3.2. Tracking Based on Correlation Filtering

In basic cases, a basic template matching algorithm can perform target tracking; however, with template-based filtering, the detector’s response value to the background is likewise large, and the capacity to distinguish between the target and the background is insufficient. The design philosophy in a wave device is to provide the highest possible reaction in the target position while producing the lowest possible response in the background position. By identifying the peak of the input picture’s response through the filter, we can accurately predict the target location.

The convolution theorem is the basic principle of correlation filtering tracking.

(9) $$f\left(x,y\right)⊗h\left(x,y\right)\Leftrightarrow F\left(u,v\right)⊙H\left(u,v\right)$$

**Figure 6:**
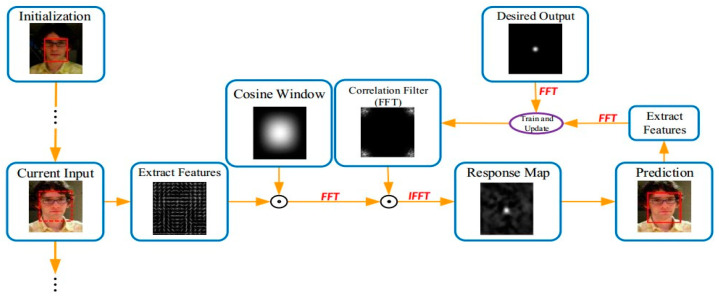
Typical correlation filter tracking algorithm flow.

Here, it can be shown that determinations are made by multiplying the matrix in the frequency domain and then returning it to the spatial domain using an inverse Fourier transform. We extract the features in the current frame and then use a cosine window to remove the window border’s discontinuity. We then perform a Fourier conversion of the feature’s value into the frequency domain, followed by a correlation operation using the correlation filter to generate the frequency domain. To obtain a spatial response, we use the inverse Fourier transform; the spatial response’s maximum point is the projected target position. Finally, depending on the characteristics of the projected position and the standard output retrieved, they are combined to generate a training sample for a correlation filter. The following Figure 6 shows the mecanism of a typical correlation filter tracking algorithm flow.

The mechanism for determining the filter is at the heart of correlation filtering. To use the nuclear correlation filtering tracking algorithm, first, regression must be conducted; then, training with the circulant matrix sample set is required; and finally, the correlation filter is obtained.

### 2.4. KCF Tracker Size Estimation

**Figure 7:**
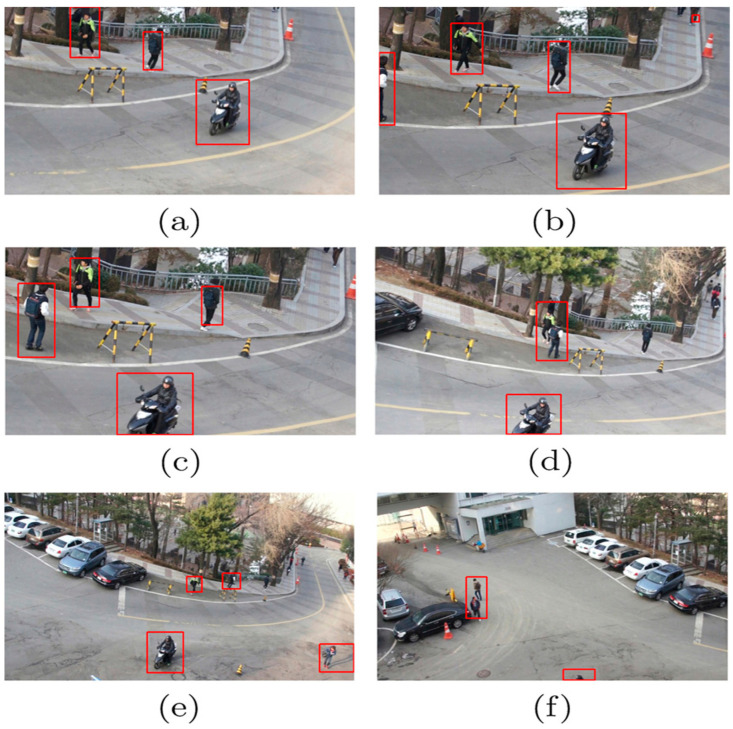
KCF tracking with target scale changing.

Since the nuclear correlation filter can only output the target’s position during tracking, it cannot adapt to alterations in the target’s scale during the tracking process. As a result, when the goal’s scale varies significantly throughout the tracking process, the standard KCF fails. This leads to a significant reduction in the tracking accuracy of the algorithm. The results of the KCF are shown in the middle two images of video collection of different targets’ scale shown in Figure 7. During the algorithm test, we discovered that when the targets’ scale significantly changes from 7(a) to 7(b), the system cannot adapt to the changes in the targets’ size, and tracking drifts.

On the one hand, many existing detection-based tracking algorithms only estimate a target’s translational location without accounting for changes in scale. Some current approaches, on the other hand, use scale estimates, but their tracking rates are quite poor; as a result, given the research is based on the KCF, it is important to calculate the size of the KCF. The algorithm framework’s excellent computational efficiency can assure rapid tracking. On the other hand, improving the scale estimates can improve the tracking accuracy and stability.

Because nuclear correlation tracking filtering is used to tackle the problem of changes in target scale, the scale estimation is more accurate, and a scale estimation approach based on the scale pyramid search space is provided.

### 2.5. Long-Term Tracking Research Based on Kernelized Correlation Filtering

If the target tracking algorithm is to accomplish long-term tracking, many main concerns must be addressed: the algorithm must address several key concerns, including motion suppression of template drift, long-term occlusion of the target, the target moving out of the field of vision, and movement of the target or camera [9]. The suggested approach can handle short-term occlusion of the target but not long-term occlusion, and because the detector does not conduct a global search, the object cannot be identified again once it has moved out of the field of vision [15]. In the proposed tracking learning detection (TLD) framework, tracking and detection happen simultaneously; the tracker generates training samples to train the detector, reinitializing it when the tracker malfunctions [18]. On the other hand, TLD tracks and performs inspection tests concurrently, resulting in a slower algorithm execution rate. We propose an algorithm framework that integrates tracking and detection utilizing the kernel correlation filter (KCF) target tracking method. We analyze the tracking failure process and propose a tracking confidence index for use during the tracking process. We identify the current tracking conditions and modify the tracking approach based on our confidence in the peak-to-sidelobe ratio (PSR) [13]. If the PSR falls below the threshold, the KCF model judges the tracking to have failed, halts the update, and switches the detection module to search for the target position in the global scope. The detection module must search for and place targets within the overall picture. To improve the real-time performance, it is necessary to employ a cascade detector. In the first stage, the variance classifier is used to filter out variance values less than 50% of the target variance value.

#### 2.5.1. Tracking Based on Evaluating Confidence in the PSR of the Tracker

**Figure 8:**
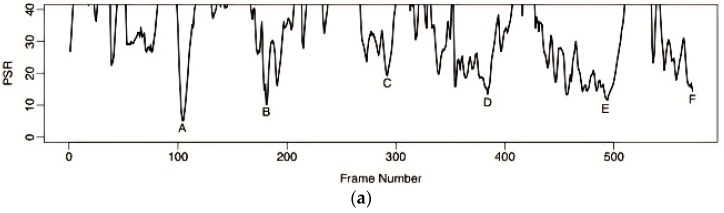

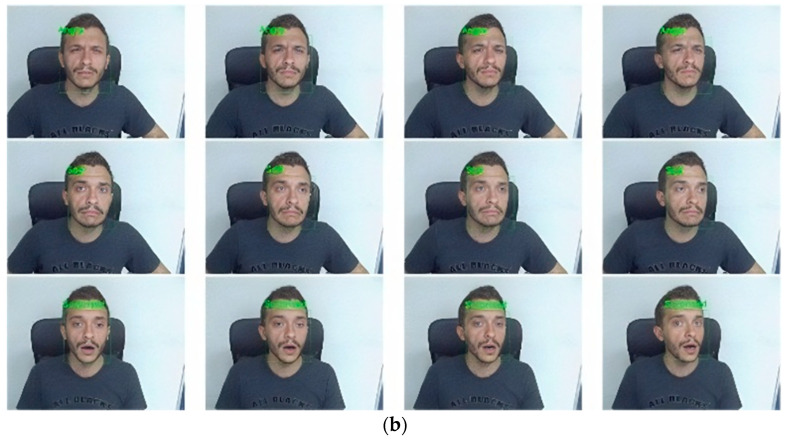
KCF tracking of the PSR in a sequence of low-level images of Dudek’s facial expressions.

Under typical tracking conditions, the identified picture block passes the KCF. The filter operation results in a fairly high spatial distribution, and the peak in the output picture corresponds to the anticipated target location. As a result, we can utilize its degree of strength to define the tracker’s level of confidence. MOSSE [19] is an abbreviation for Minimum Output Sum of Squared Error, which was discovered in the PSR algorithm for properly assessing the peak intensity. The PSR formula is given by [13,20]:

(10) $$PSR=\frac{g_{max}-\mu_{x_{1}}}{\sigma_{x_{1}}}$$

where *g_max_* is the maximum value of the response map, an 11 × 11 window is selected around the peak corresponding to the maximum value, and $\mu_{x1}$ and $\sigma_{x1}$ are the average value and standard deviation of the response map in the window, respectively. From Equation (10), we can see that the PSR calculates the relative value of the peak value and the side value. When the face tracking situation is favorable, the PSR is relatively high, and the location of the response peak represents the new position of the face. Conversely, a lower PSR indicates that a face is obscured. Testing the algorithm on a test video set revealed a typical range of PSR values during tracking of 20 to 60. A simple case analysis was performed on a video series, as illustrated in Figure 8b, where our volunteer Dudek expressed his feelings during KCF tracking, with the distribution of the PSR measured throughout the sequence. The diagram indicates that the level of the PSR at points A, B, C, D, E, and F was relatively low. According to the results of the analysis (see Figure 8a), these points correlate with the target’s occlusion, distortion of his appearance, quick movements, rotation, him leaving the field of vision, and occlusion. As a result, the PSR seems quite good; this value reflects the tracker’s tracking performance.

#### 2.5.2. The Variance Classifier

**Figure 9:**
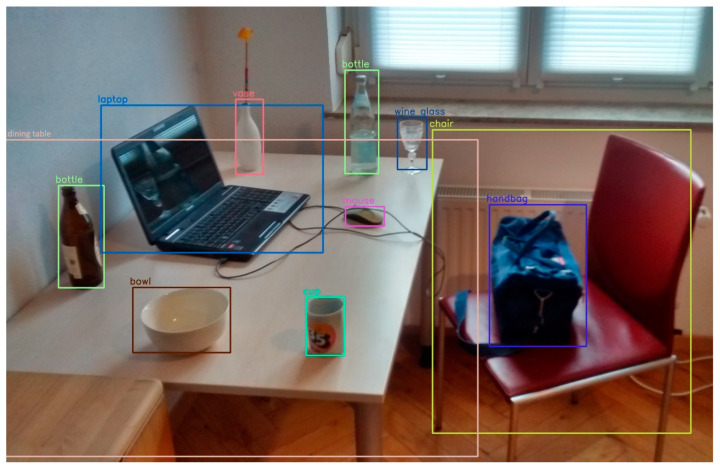
Stage detectors for a variance classifier applied to an image to obtain and filter background information.

The detector here is made up of two-stage detectors. The cascade classifier must be able to swiftly filter out regions that do not contain the target for the first-stage classifier. After this first screening, the more accurate classifier is applied to detecting the remaining target frames, which can save a significant amount of calculation time. Figure 9 illustrates the classification of the blue-labeled images into background areas, where, intuitively, they have almost no presence; however, the detailed information reveals slight variance. The yellow-labeled areas are high-variance areas, and their details are rich.

The variance in the target frame is calculated in the following through an integral graph. By definition, *x* is the first value in the target frame. Its value can be determined by Formula (11), which defines the variance:

(11) $$\sigma^{2}=\frac{1}{n}\sum_{i=1}^{n}{\left(x_{1}-\mu \right)}^{2}$$

where *n* is the number of pixels in the target frame, and µ is the average level of gray in the pixels in the target frame, which can be determined using the following formula, (12):

(12) $$\mu =\frac{1}{n}\sum_{i=1}^{n}x_{i}$$

Formula (11) can be transformed into the following formula:

(13) $$\sigma^{2}=\frac{1}{n}\sum_{i=1}^{n}{\left(x_{1}-\mu^{2}\right)}^{2}$$

A target frame of pixels requires n memory query operations for the variance to be determined, but the integral graph technique requires just eight memory query operations. The integral graph *I* and the source picture *I* are the same size, and the integral graph is in the coordinate system (*x*, *y*). The original picture (Figure 1) is represented by the value of the level of gray in (*x*, *y*)’s pixels between areas. Formula (14) provides the definition of the integral graph:

(14) I(x,y)=∑I(x,y)

The integral graph allows for rapid determination of the total gray values in the rectangular region. For example, for a rectangle *B* (*x*, *y*, *w*, *h*), the coordinate value of the top-left corner of the rectangular box is *x*, *y*, and the width and height of the rectangular box are *w*, *h*. For a B-shaped rectangle, the sum of the values of its inner pixels is

(15) $$\sigma^{2}=\frac{1}{n}I(B)-[1/n-I(B)]$$

### 2.6. The Tracking Algorithm Framework Combined with Detection

If the target tracker fails in its tracking, we switch to the target detection mode and perform target detection throughout the entire picture. Target tracking does not start until the target is identified. The tracking algorithm used by the KCF is based on a measurement tracking algorithm; the tracker in question is a detector located in a small region close to the target location in the previous frame which performs target processing across the entire picture. The two are very similar. However, the detection area differs.

When the target is constantly moving, utilizing the tracker might result in a quicker tracking rate. When the target is blocked for an extended period of time, the target’s position can still be recognized in the global scope. A cautious target model updating technique can be used to prevent drift of the target model while retaining a description of the target’s most recent form.

**Figure 10:**
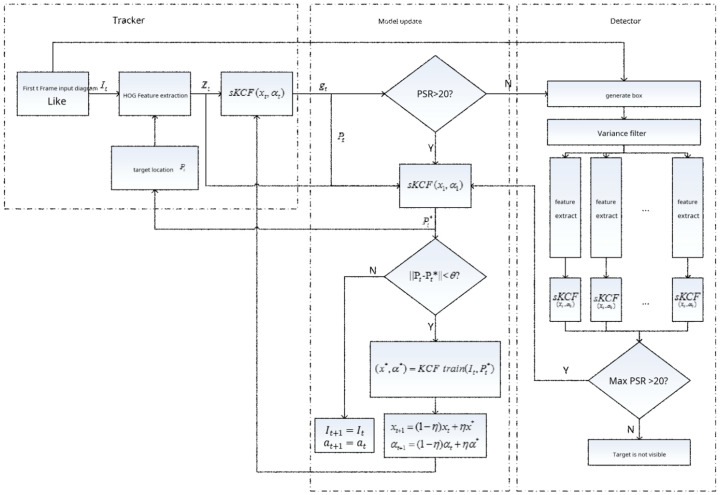
Flow chart of tracking algorithm combined with detection.

Figure 10 illustrates the framework for the entire algorithm.

TLD tracks and detects at the same time. Checks are only used in the context of this article when the tracker fails in tracking. The detector detects the target at the global scale. Furthermore, the tracker and the detector employ the same KCF model as the target model. The goal is to differentiate the target from its surroundings. The current frame’s tracker is dependent on the target location monitored in the previous frame. This process involves extracting the features of histograms of oriented gradients (HOGs). We scale the detection feature vector Z_t_ using an sKCF and calculate the filter’s response using g_t_. We can estimate the PSR based on the response g_t_.

If the PSR is greater than 20, the tracker’s results are deemed reliable, and the model is updated. If the PSR is less than 20, the link is activated. It is assumed that the tracker failed in tracking, leading to the following outcomes: the first scenario involves changes in the target’s appearance, such as occlusion or distortion, while the second scenario involves the target or the camera moving violently and undergoing rapid changes. Consequently, we are unable to locate the target in the previous frame’s vicinity in these cases. At this point, we start global target detection to determine the target’s position.

If the tracker’s PSR is greater than 20, the algorithm refreshes the model and indicates that the tracking is normal. To begin with, using the first frame of the model retrieved at the maximum response of the tracker P_t_, we obtain the feature vector Z’. The target model Z’ is referred to in the first frame, and we apply linear filtering to obtain the maximum response position (P_t_*). If this ||P_t_ − P_t_*|| is less than a given threshold, the model can be updated. We obtain I_t_ and P_t_* during KCF model training, and we determine α_t+1_ and x_t+1_ as follows:

(16) αt+1=1−ηαt+ηαt+1xt+1=1−ηxt+ηxt+1

where ƞ represents the learning rate, tuned to a small value in practice. 

If the tracker’s PSR is not greater than 20, the algorithm activates the detection module. For target detection, the detection module examines all candidates in the supplied picture location. First, the variance in the gray value is less than the initial variance in the goal value thanks to the variance classifier. To speed up the calculation, half of the target frame to be identified is filtered out. This strategy is effective for tracking situations with a basic background because the variance classifier can exclude most basic backdrops, such as blue sky and white clouds. On the passing side, the difference classifier recognizes the sKCF in the target frame, carries out target detection, and responds to all the target boxes using the PSR values.

When the PSR exceeds 20, this signifies the presence of the target in a box. The model update module then receives the detected location and updates the model accordingly. If the maximum PSR value is less than 20, this indicates that the detector did not detect the target. The target has not yet appeared in the picture, and the target detection module will continue to run to the next frame. The detected target position is used to reinitialize the tracker.

This section provides an algorithm framework that integrates the nuclear correlation filtering tracking method with tracking and detection. We monitor tracking failures based on confidence. When the PSR falls below the threshold, we judge the tracking to be unsuccessful, halt updates to the KCF model, and switch the detection module to search for the target position in the global scope. The detection module employs a cascade detection strategy.

First level: We use a variance classifier to filter out variance values that are less than 50% of the desired variance value.

Second level: We use the nuclear correlation filter to perform target detection on the sample that passes the first detector during the test. The target becomes visible in the current frame if the maximum confidence exceeds the threshold, and the location with the highest confidence among these samples serves as the projected target location; otherwise, the target remains invisible in the current frame.

## 3. Experimental Results and Analysis

### 3.1. The Algorithm Evaluation Index and Test Dataset

#### 3.1.1. The Algorithm Evaluation Index

The center location error (CLE) is a commonly employed evaluation metric for tracking precision. It is defined as the average Euclidean distance between the center positions of tracked targets and the manually annotated ground truth. The overall performance of a sequence is then determined by averaging the center location error over all of its frames. Nevertheless, when the tracker fails to maintain the target, the resultant location may be arbitrary, and the average error value may not accurately reflect the tracking efficacy.

Analysis of the tracking algorithm focuses on two primary aspects: tracking accuracy and tracking robustness. We classify tracking robustness as either robustness to time or spatial robustness. Wu Yi came up with a form of tracking algorithm evaluation [21] which uses the distance precision (DP) and the success rate (SR) to measure how well the tracking works. The distance precision (DP) is adopted to measure the overall tracking performance; the distance plot shows the percentage of frames in which estimated location is within the given threshold distance of the ground truth. To measure the performance on a sequence of frames using the success rate, we count the number of successful frames whose overlap S is larger than the given threshold t_o_. The success plot shows the ratios of successful frames at the thresholds varied from 0 to 1. Using one success rate (SR) value at a specific threshold (for example, t_o_ = 0.9) may not be fair or representative for tracker evaluation. Instead, we use the area under curve (AUC) of each success plot to rank the tracking algorithms. One-pass evaluation (OPE), spatial robustness evaluation (SRE), and temporal robustness evaluation (TRE) are used to measure how robust the tracking is.

**Figure 11:**
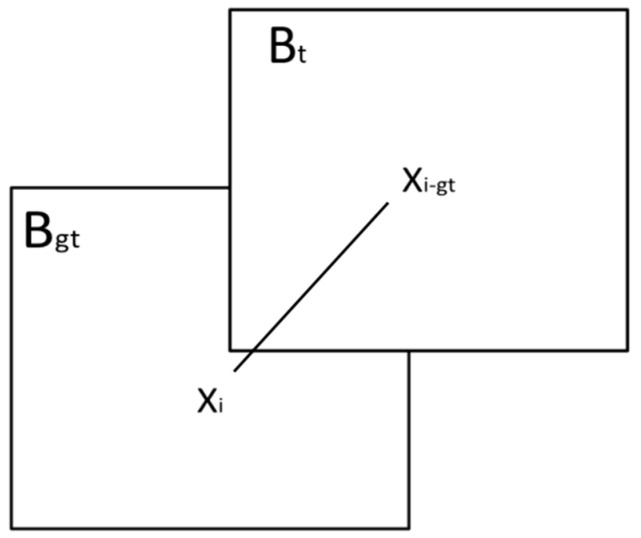
Schematic diagram of CLE and overlap of the tracking effect in a single frame.

The center location error (CLE) and the degree of overlap (S), shown schematically in Figure 11, are the two main ways to measure the tracking effect in a single frame. The error is defined as the Euclidean distance between the target’s central position (*x_i_*, *y_i_*), obtained by the tracker, and the target’s true position (*x_i_*_-*gt*_, *y_i_*_-*gt*_), annotated by the dataset, as in Equation (17). The intersection of the two sets is the degree of overlap between the bounding box and the explicitly selected target bounding box (see Figure 11). In each case, (17) and (18) provide the mathematical formula for the CLE and the overlap *S*:

(17) $$CLE=\sqrt{{\left(x_{i}-x_{i-gt}\right)}^{2}+{\left(y_{i}-y_{i-gt}\right)}^{2}}$$

where (*x*, *y*) indicates the target position output by the tracker, and (*i*-*gt*) indicates the manually marked target position.

(18) $$S=\frac{\left|B_{t}\cap B_{gt}\right|}{\left|B_{t}\cup B_{gt}\right|}$$

The variables *B_t_* and *B_gt_* represent the tracker’s target bounding box and the manually marked target bounding box (the ground truth bounding box), respectively.

We cannot adequately evaluate the tracking effect using the CLE with overlap and the average value approach because the tracker’s output is relatively unpredictable when it loses the target, and when it is affected by overlap, the CLE is worthless. Formula (19) formalizes this paraphrasing and calculates the distance precision (DP) to evaluate the tracker [12,13]. Formula (19) yields the number of video frames below a specified threshold (as the representative precision score for each tracker, we use the score for a threshold of 20 pixels), with m representing the number of video frames that do not meet the given threshold, while n denotes the total number of video frames.

(19) $$DP=\frac{m}{n}$$

In [11], Wu Yi mentioned that the SR (success rate) assesses the tracker’s overall performance, which is similar to the DP (distance precision).

The standard tracker evaluation method involves running trackers through a test sequence, initializing them from the ground truth position in the first frame, and reporting their average performance using the DP and SR to evaluate and analyze their results. This method is known as the single assessment approach, or one-pass evaluation (OPE). However, individual trackers may be sensitive to initialization, and initialization from various frames may also affect the tracker performance and OPE. The model does not reflect these variations in the trackers. We employ both spatial robustness evaluation (SRE) and temporal robustness evaluation (TRE). Upon initializing the tracker, we offset the artificially designated bounding box in the first frame by a specified degree in each direction, using both the offset bounding box and TRE. The tracker initializes distinct frames in the video as the first frames and then starts tracking the targets in each frame from that point on.

In real-world applications, an object detector typically initializes a tracker, which may include initialization errors regarding location and size. Furthermore, an object detector can reinitialize a tracker at various time intervals. In robustness evaluation, investigating a tracker’s characteristics allows for a more comprehensive understanding and study of the tracking method.

Upon receiving a beginning frame and the corresponding ground truth bounding box of the target, the SRE initializes a tracker, which continues to operate until the conclusion of the segment within the entire sequence. The tracker is assessed on each segment, and the cumulative statistics are compiled.

To obtain the initial bounding box for the TRE, we adjust or resize the ground truth in the first frame. We employ eight spatial shifts, four center shifts, four corner shifts, and four scale variants (supplement). The amount of shift is 10% of the goal size, and the scale ratio varies between 0.8, 0.9, 1.1, and 1.2 relative to the ground truth. Consequently, we assess each tracker a total of 12 times for the SRE.

#### 3.1.2. The Test Dataset

**Figure 12:**
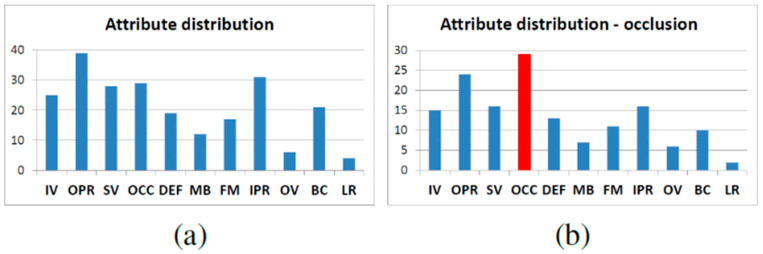
(**a**) Attribute distribution of the entire test set and (**b**) distribution of the sequences in terms of the occlusion (OCC) attribute [20].

As the research on target tracking algorithms has progressed, numerous institutions have gathered video collections for researchers to test on and compare, ensuring fair and unbiased studies of algorithms. For example, in the field of video surveillance, VIVID [14] collaborates with CAVIAR [15] and a broader video collection, while David et al. [18] collaborate with Babenko et al. [19]. Researchers have collected and marked 50 video sequences, assigning 11 different types of attributes to them. In these annotated video test sets, the researchers labeled each sequence’s properties, simplifying comparisons and assessments of different trackers’ performance under various characteristics. These 11 attributes are illumination variation (IV), deformation (DEF), scale variation (SV), occlusion (OCC), motion blur (MB), fast motion (FM), in-plane rotation (IPR), out-of-plane rotation (OPR), out-of-view (OV), background clutter (BC), and low resolution (LR) [22]. Furthermore, we can assign multiple qualities to each video set simultaneously, and these qualities are not mutually exclusive as can be seen in the following Figure 12.

Moreover, a sub-test set with unique properties can also be used to test the method on the full test set. We examine the tracking algorithm to explore how different algorithms adapt to varied settings.

### 3.2. Analysis of the Algorithm’s Improvement Effect

#### 3.2.1. Overall Comparison of the Effect 

**Figure 13:**
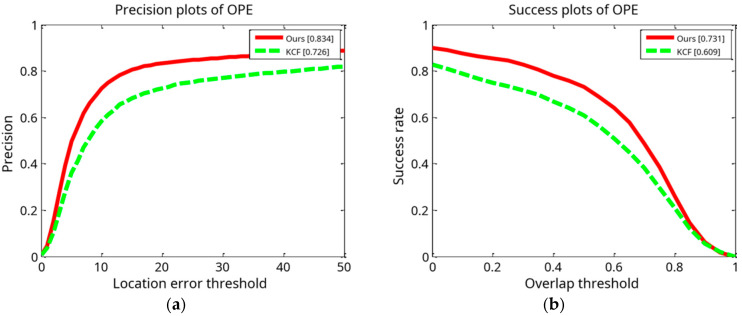
Results of tracking experiment carried out on two test video sets: (**a**) precision plot of OPE and (**b**) success plot of OPE.

**Figure 14:**
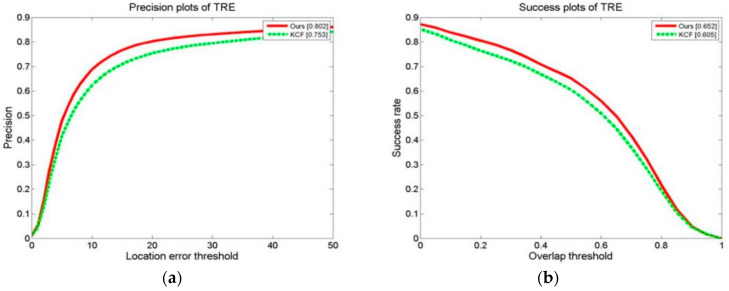
Results of tracking experiment carried out on two test video sets: (**a**) precision plot of TRE and (**b**) success plot of TRE.

**Figure 15:**
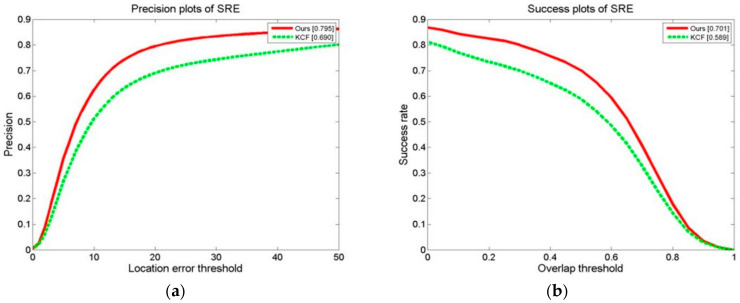
Results of tracking experiment carried out on two test video sets: (**a**) precision plot of SRE and (**b**) success plots of SRE.

We conducted a tracking experiment on two test video sets, as shown in Figure 13a,b, with OPE (one-pass evaluation). They show the distance precision (DP) on the left and the success rate (SR) on the right during the experiment. Figure 14a,b display the temporal robustness evaluation (TRE) results, displaying the DP on the left and the SR on the right. Figure 15a,b display the SRE (spatial robustness evaluation) results, with the DP on the left and the SR on the right. The precision plots in the images and the numbers in the figure legends denote the CLE, with a position error of less than 20 degrees.

The success plot is the ratio of the number of frames per pixel to the total number of frames. In the images, the legend number denotes a greater overlap than the ratio of the number of frames to the total number of frames [1]. During the experiment, this paper’s method’s DP of 0.834 with a KCF of 0.726 improved the algorithm by 14.9%. This paper’s method’s SR of 0.731 with a KCF of 0.609 improved the algorithm by 20.0%. For the robustness test in relation to time (TRE), this paper’s method’s DP of 0.802 with a KCF of 0.753 improved the algorithm by 6.5%. This paper’s method’s SR of 0.652 with a KCF of 0.605 improved the algorithm by 7.7%. For the robustness test in terms of space (SRE), this paper’s methodology obtained a DP of 0.795 with a KCF of 0.690. This paper’s method’s SR of 0.701 with a KCF of 0.598 improved the algorithm by 17.2%.

This study employed a method that significantly increased the value of the KCF in every test scenario. The algorithm significantly improved the tracking accuracy, and the TRE testing shows that this study’s method had no impact on the OPE, thereby confirming the efficiency of the KCF. Although the accuracy of the impact of short-term tracking was strong, the impact of short-term tracking was not explicitly increased because of the TRE in this article. When the video sequence diverges, the tracking algorithm initiates and completes follow-up tracking at a specific moment. This results in an average video within the entire video set with a frame length less than that in the OPE. Meanwhile, we halve the SRE. We introduce disturbances of various orientations and scales near the manually indicated target location during the test on purpose, as background information is easier to introduce at this location, reducing the likelihood of drift during the tracking process. When the test findings are related to those of OPE, they do not change considerably, showing that the algorithm and the KCF are effective. The algorithm’s anti-drift capabilities are robust.

#### 3.2.2. Comparison of Video Tracking Effects with Scale Conversion

The CarScale subsection of this research uses a case study of test videos, as per the KCF, to verify the validity of the scale transformation algorithm. Furthermore, this paper comprehensively compares the methods employed. The size of the target automobile fluctuates dramatically throughout the test video sequence. In a subsequent frame, the car’s area is 34.5 times its value in the first frame. The automobile’s size in the first frame is 42 × 26 pixels, and the maximum size throughout tracking is 306 × 123 pixels. We implement the algorithm on the video set in a CarScale tracking situation, with green frames representing the target location estimates provided by the research technique and red frames representing those of the KCF, which provide an estimate of the target’s position. As observed in the tracking process, the algorithm’s intended bounding box does not change in size and cannot adjust to variations in the car’s scale. However, the addition of a scale pyramid search strategy allows the algorithm in this study to track even the smallest changes in the automobile’s size, potentially providing a reasonable estimate of the target’s scale.

**Figure 16:**
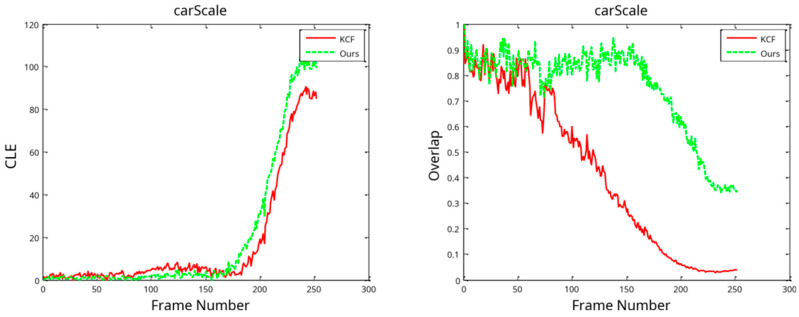
Tracking process with the center location error (CLE) and overlap distribution.

Figure 16 illustrates the tracking process using the center location error (CLE) and the overlap distribution. The distribution diagram demonstrates its adaptability to changes in the target’s scale, resulting in a significantly larger overlap than that of the KCF. However, in this study, this method’s center location error is higher than that of the KCF in the distribution diagram by a significant margin. This is because despite the target’s scale not being estimated, the target’s output position consistently lies in the vehicle’s center, resulting in a minimal center position error. Additionally, the methodology’s scale estimation alters the car’s scale due to the occlusion of a small number of trees in the video sequence during the occlusion procedure. As a result, the CLE in the algorithm’s determination of the car’s position is somewhat inaccurate. According to the graph, this study’s methodology is somehow less accurate than the KCF, but the benefits of a scale estimate for the target outweigh the disadvantages of the low accuracy in determining the center position.

#### 3.2.3. Analysis of the Effect of the Model Update Strategy

**Figure 17:**
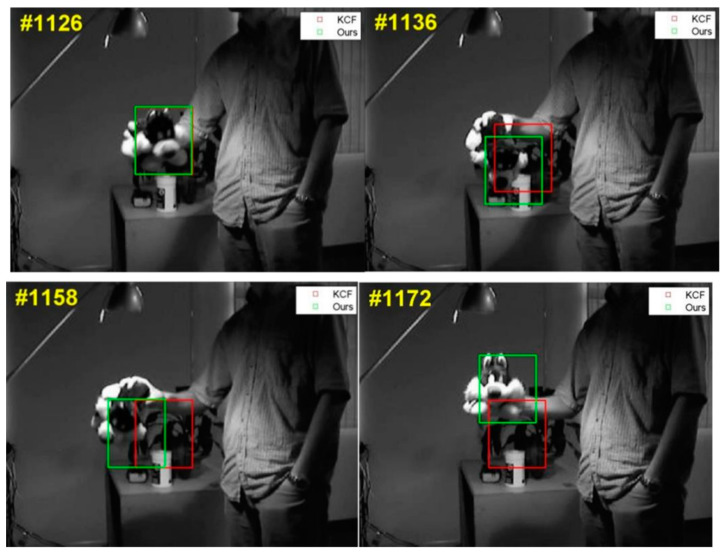
Effect in tracking a video set of Sylvester.

Figure 17 illustrates a tracking scenario on the video set, with the green frames representing the target position estimates provided by the algorithm in this research and the red frames representing those of the KCF. During the target tracking process, one can observe that the target has a wider angle of view compared to the camera, leading to dramatic variation in the target’s appearance during changes in the goal due to the KCF. Target drift occurs when a basic model update approach incorporates a large amount of background information into the target model. This study employs a methodology that reduces the model drift by utilizing the model information from the first frame, thereby minimizing the introduction of background information.

**Figure 18:**
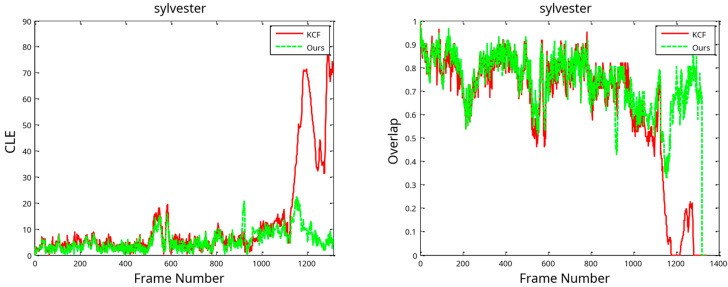
Results of tracking process on Sylvester videos in terms of CLE (**left**) and overlap distribution (**right**).

Figure 18 illustrates the CLE with the overlap in the tracking process. The methods’ outcomes are extremely close when the change in the target is not significant; however, when the target varies greatly, the KCF method’s tracking fails, but the algorithm in this work can still follow the target successfully. So, based on the results of tracking the Sylvester video set, the model update technique presented in this study may successfully handle the problem of model drift in the tracking process.

#### 3.2.4. Comparison of the Effect of Target Occlusion in Video Tracking 

**Figure 19:**
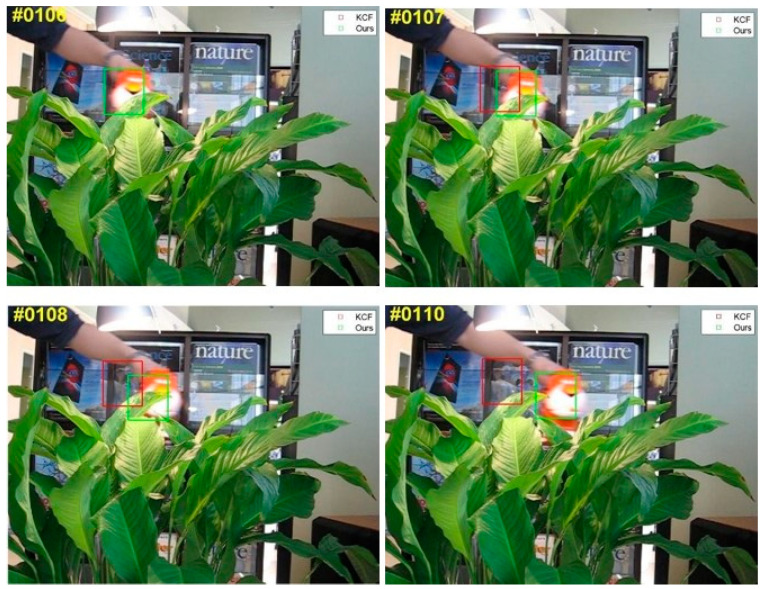
Tracking results of two trackers (the KCF and ours) on Tiger2.

In Figure 19, the algorithm tracking scenario is shown for a video set of Tiger2, with the green frames representing the target position estimates provided by the method in this research and the red frames being the actual target’s location. These images display the algorithm’s predicted target positions, suggesting that the target moves to the right during the tracking procedure. The target is harder to locate when it is moving.

When an algorithm comes up against occlusion, relatively basic model updating techniques inject background information into the algorithm’s target model, leading to tracking failure. Meanwhile, the algorithm in this study has the potential to effectively use a model updating technique. To overcome the target occlusion problem, it avoids injecting background information into the target model. Even if the target is partially obscured, the method used in this study can detect it with the cascade detector once it returns to the field of vision. The tracker is then reset to guarantee continuous tracking of the target.

**Figure 20:**
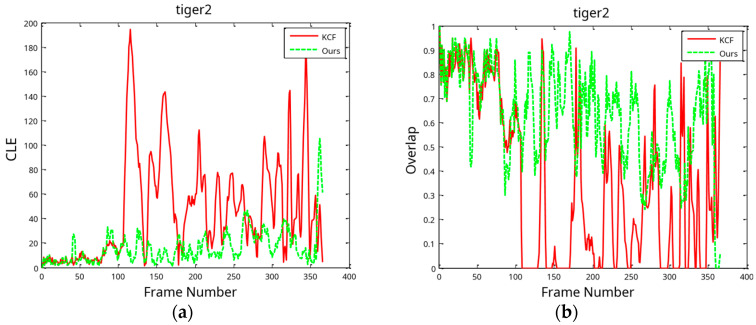
Tiger2 tracking results in terms of CLE in tracking process (**a**) and overlap distribution (**b**).

Figure 20 illustrates the CLE in the tracking procedure and the overlap distribution. This work uses a method that maintains a decent tracking state throughout the tracking process, unlike the KCF. The latter algorithm made frequent hops, resulting in numerous time overlaps equal to 0. This example indicates failure to achieve the intended objective. The preceding test’s results indicate that the model updating technique in this research effectively injects information into the target model, thereby solving the target occlusion problem. However, the KCF target tracking is susceptible to drift when the algorithm handles an obstructed target.

### 3.3. Comparative Analysis of the Algorithm in This Paper and Other Algorithms

#### 3.3.1. The Overall Effect

This section describes the use of KCF Algorithmic 9. We tested the tracking algorithm library on 50 video sets, including the target tracking algorithm and the method described in this work. This was the configuration of the experimental platform in this article: CPU: IntelI Core I i5-4590; 3.30 GHz; RAM: 16 GB; graphics card: NVIDIA GeForce GTX660.

Attributes are organized into columns in the following table. In the Feature Representation column, HOG stands for histogram of directional gradients, L for local characteristics, H for holistic characteristics, SR for sparse representation, BP for binary pattern (characteristics of binary modes), and SPCA for sparse principal component analysis. In the Target Classifier column, DM represents a discriminative model, while GM represents a generative model. Regarding the search methodology, PF stands for particle filtering, a term that refers to the process of filtering particles; LOS stands for local optimum search; MCMC stands for Markov Chain Monte Carlo; and DS stands for the dense sampling approach. In the Code Form column, M refers to MATLAB, C refers to C/C++, and E represents executable binary code.

Table 1 displays the RS, DP, and FPS indexes of the algorithms included in the evaluation. Among these, the success rate of the algorithm from this article has an RS value of 0.726, the highest among all the algorithms, and the KCF algorithm has an RS value of 0.607. The algorithm used in this work has a DP value of 0.829, which is the highest among all the algorithms, followed by the KCF algorithm, which has a DP value of 0.716. The KCF has the greatest frame rate of all the algorithms in the assessment, with an average frame rate of 200.3 frames per second. This study’s method achieves the second highest frame rate, clocking in at 50.1 frames per second. In general, the method described in this work is a KCF. However, when compared to other methods, the approach in this study outperforms them in terms of its tracking performance.

**Figure 21:**
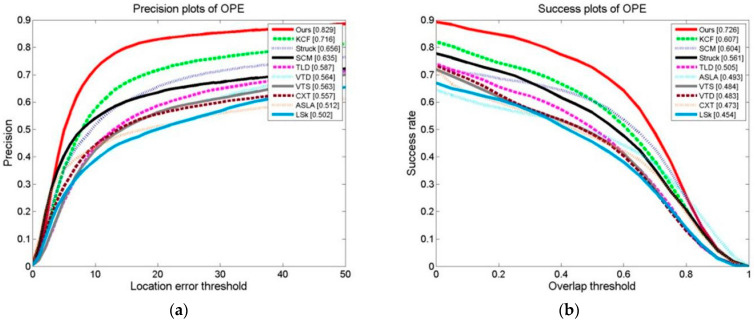
Results of OPE of the tracking algorithms on 50 video collections: (**a**) precision plot of OPE and (**b**) success plot of OPE.

**Figure 22:**
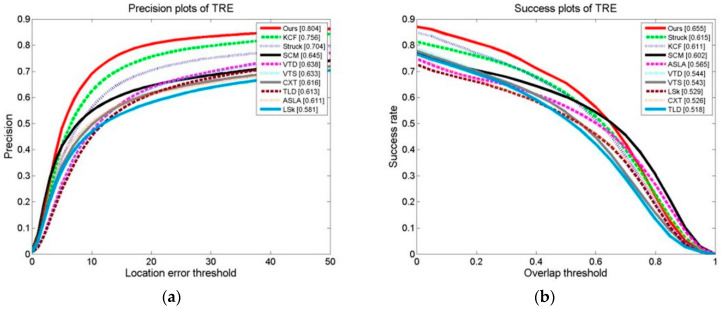
Results of TRE of the tracking algorithms on 50 video collections: (**a**) precision plot of TRE and (**b**) success plot of TRE.

**Figure 23:**
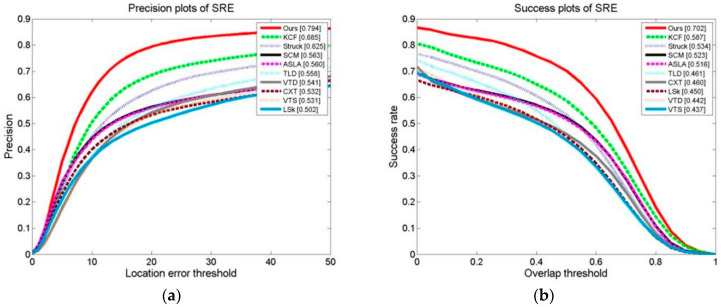
Results of SRE of the tracking algorithms on 50 video collections: (**a**) precision plot of SRE and (**b**) success plot of SRE.

OPEs, SREs, and TREs of the tracking algorithms were conducted on 50 video collections. As can be seen, Figure 21, Figure 22 and Figure 23 demonstrate that the algorithm in this paper significantly outperformed the other algorithms on all of the test content, with the KCF algorithm following closely behind.

#### 3.3.2. Comparison of Tracking Algorithms under the Scale Conversion Test Set

**Figure 24:**
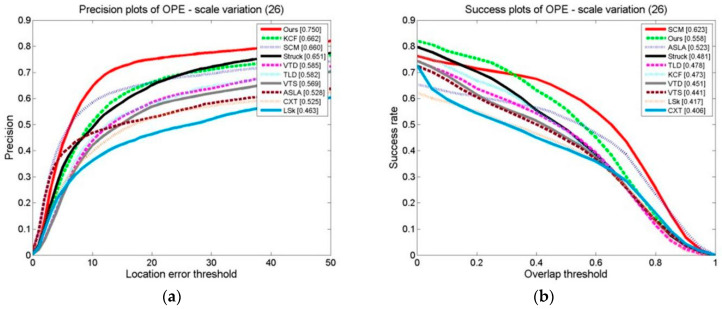
OPE of tracking algorithms on target scale variation sets: (**a**) precision plot of OPE and (**b**) success plot of OPE.

**Figure 25:**
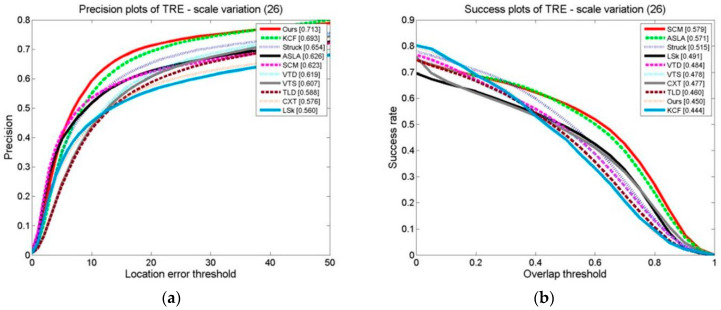
TRE of tracking algorithms on target scale variation sets: (**a**) precision plot of TRE and (**b**) success plot of TRE.

**Figure 26:**
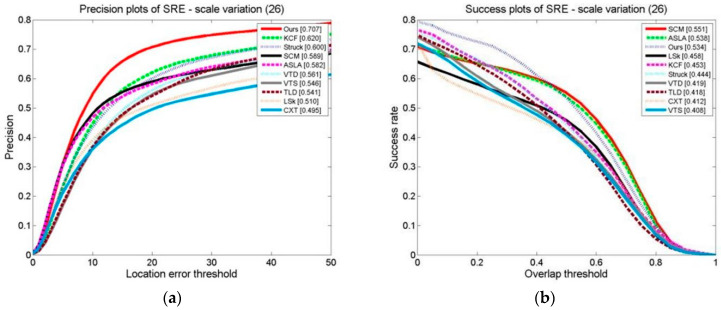
SRE of tracking algorithms on target scale variation sets: (**a**) precision plots of SRE and (**b**) success plot of SRE.

The OPE, TRE, and SRE of the tracking algorithms on the scale change video sets are shown in Figure 24, Figure 25 and Figure 26. The distance precision (DP) and success rate (SR) metrics are used. The figures demonstrate that the algorithm significantly outperforms the other algorithms when using the DP as the evaluation index. When the target’s scale changes, the algorithm has a higher tracking distance accuracy. However, when using the SR as an evaluation index, the algorithm obtains slightly lower results than the SCM algorithm, ranking second. The SCM algorithm estimates the target scale well and the target’s position precisely.

#### 3.3.3. Comparison of Tracking Algorithms on a Target Occlusion Test Set

**Figure 27:**
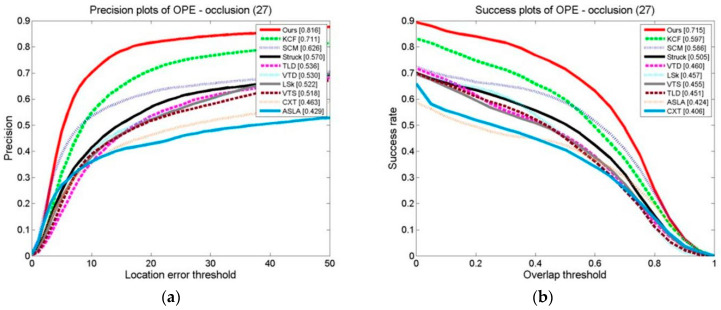
Tracking algorithms on the target occlusion video sets: (**a**) precision plot of OPE and (**b**) success plot of OPE.

**Figure 28:**
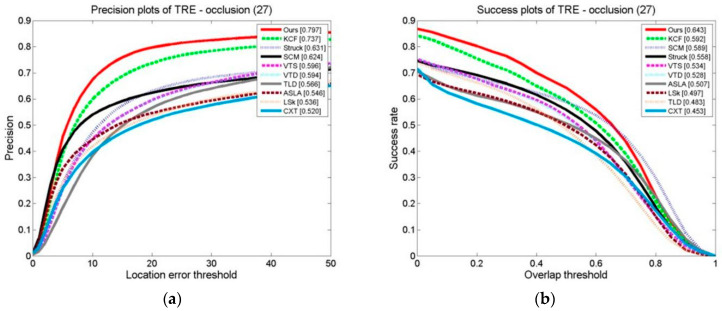
Tracking algorithms on the target occlusion video sets: (**a**) precision plot of TRE and (**b**) success plot of TRE.

**Figure 29:**
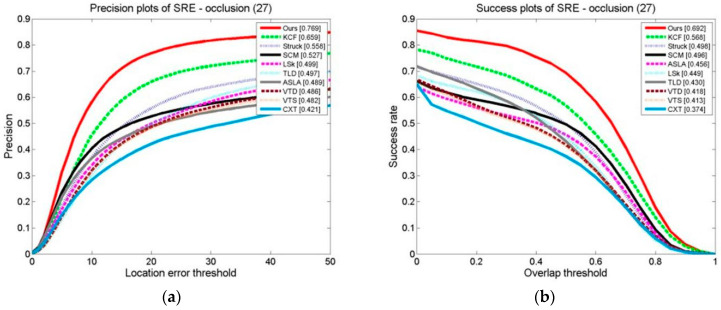
Tracking algorithms on the target occlusion video set: (**a**) precision plot of SRE and (**b**) success plot of SRE.

The results of ten formalized examples of tracking algorithms on a target occlusion video set are shown in Figure 27, Figure 28 and Figure 29. These include OPE, TRE, and SRE, using the distance precision and success rate. The graphs illustrate whether we have used the DP as the assessment index or based it on the SR.

In terms of the evaluation indexes, our algorithm outperforms the others. This demonstrates that the method suggested is superior to the other algorithms in terms of flexibility when a target is obstructed. This is because the kernel correlation filtering technique is particularly strong in distinguishing between the scene and the target, and the model updating technique suggested can effectively prevent the introduction of the target model, which stops model drift.

#### 3.3.4. Comparison of Tracking Algorithms on a Test Set in Which the Target Exits the Field of View

**Figure 30:**
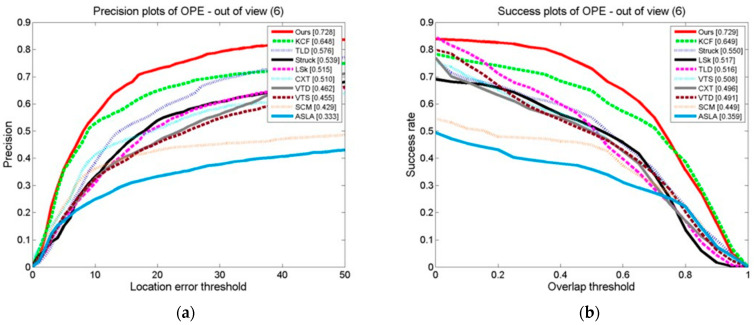
The tracking algorithms on the test set with the target out of view: (**a**) precision plot of OPE and (**b**) success plot of OPE.

**Figure 31:**
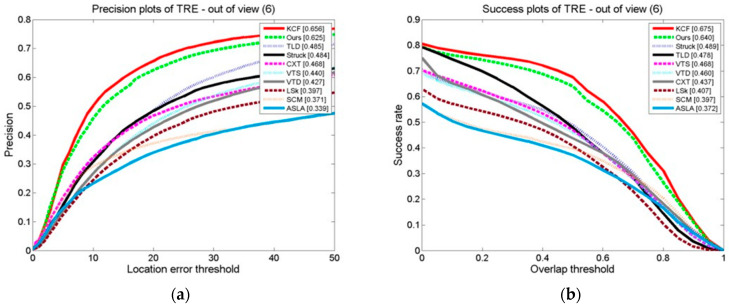
The tracking algorithms on the test set with the target out of view: (**a**) precision plot of TRE and (**b**) success plot of TRE.

**Figure 32:**
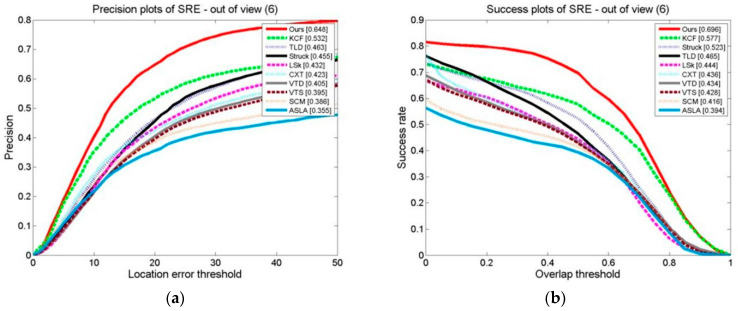
The tracking algorithms on the test set with the target out of view: (**a**) precision plot of SRE and (**b**) success plot of SRE.

Ten examples are shown in the graphs in Figure 30, Figure 31 and Figure 32 of the OPE, TRE, and SRE results on a test set in which the target leaves the field of vision for tracking algorithms in terms of their DP and SRs. The graphs show that our algorithm, whether using the DP as the assessment index or the SR as the basis, outperforms the other algorithms in terms of these evaluation indexes. This demonstrates that the algorithm suggested is effective. When the target moves out of the field of view, it adapts better than the other algorithms. This is attributed to the stop model updating technique, which updates the model after marking the field of view to prevent the introduction of background information and trajectories. This is because the model updating strategy is also at a stop; the model makes updates after marking the field of view to prevent the introduction of background information and prevent causing tracking drift. When it is in the field of view, the cascade detector can effectively detect the target state and initialize the tracker.

**Interpretation:** We initially investigated a target tracking algorithm assessment system, covering the algorithm evaluation indexes and the measurements employed. The sample films covered changes in target illumination, scale, occlusion, and posture; quick motion; and challenging situations like rotation. Then, we compared the method described here to other KCFs. The overall effect on the video set, as well as the effect on tracking of changes in the scale of the target and target occlusion, demonstrates that the improvement technique suggested in this paper is an optimized KCF. Finally, we compared the results using this paper’s method, which could make KCF computations within 9 days in 50 words or fewer, making it a great tracking algorithm. Its overall effect on two video sets, as well as a comparison and analysis of its results on change in scale, target occlusion, and out-of-view target video sets, demonstrates that the method described in this study is suitable. Among the compared methods, the effect on tracking of ours is the best.

## 4. Conclusions

To solve challenges in target tracking, this work employed a two-level tracking technique, first determining the target’s coordinate location in the two-dimensional position. Then, we created a scale search space and established the ideal scale of the target within this scale space. Our experiments demonstrate that this technique is capable of resolving model drift and scale transformation issues. Complex tracking settings can obstruct targets for extended periods and render them out of view. This work proposes long-term tracking and detection which switches to the target when the algorithm detects that the tracker is not tracking effectively. The detection mode utilizes a two-stage detector to locate the target’s position within the global scope of an image.

This work offers a novel approach to real-time target tracking in complex environments using nuclear correlation tracking and detection methods. The tracking algorithm effectively balances the tracking performance and run speed. We investigated an objective assessment system, including evaluation indicators and test video sets, to evaluate the target tracking algorithm. This study objectively evaluated the proposed method and compared its performance to other existing target tracking techniques. The experimental findings validate the efficiency of the methodology presented in this study, which outperformed the existing tracking methods in terms of its tracking accuracy and tracking resilience. Overall, a real-time tracking target system based on kernel correlation filtering is a powerful and efficient tool for tracking targets and moving objects in video streams. To accurately recognize and monitor targets, target detection and tracking have been studied extensively within image-guided technology. Future research should focus on increasing the flexibility of the tracking algorithm under various settings and applying theoretical knowledge to real-world tasks.

## Figures and Tables

**Table 1:** Comparative evaluation of tracking algorithms.

Tracking Algorithm	Feature Representation	Target Classifier	Search Mechanism	Code Form	Frame Rate
Ours	HOG	DM	DS	MC	50.1
KCF	HOG	DM	DS	MC	200.3
Struck [23]	H, Haar	DM	DS	C	20.2
SCM [24]	L, SR	GM + DM	PF	MC	0.51
TLD [25]	L, BP	DM	DS	MC	28.1
ASLA [26]	L, SR	GM	PF	MC	8.5
VTS [27]	L, SPCA	GM	MCMC	MC-E	5.7
VTD [28]	H, SPCA	GM	MCMC	MC-E	5.7
CXT [29]	H, BP	DM	DS	C	15.3
LSK [30]	L, SR	GM	LOS	M-E	5.5

## Data Availability

The data presented in this study are available on request from the corresponding author for privacy reasons.
